# Maximizing Postoperative Recovery: The Role of Functional Biomaterials as Nasal Packs—A Comprehensive Systematic Review without Meta-Analysis (SWiM)

**DOI:** 10.3390/pharmaceutics15051534

**Published:** 2023-05-18

**Authors:** Rabiatul Adawiyah Razali, Ubashini Vijakumaran, Mh Busra Fauzi, Yogeswaran Lokanathan

**Affiliations:** Centre for Tissue Engineering & Regenerative Medicine (CTERM), Faculty of Medicine, Universiti Kebangsaan Malaysia, Cheras 56000, Malaysia; rabiatularzl@gmail.com (R.A.R.); ubashini.shini@gmail.com (U.V.); fauzibusra@ukm.edu.my (M.B.F.)

**Keywords:** nasal pack, functional biomaterial, wound healing

## Abstract

Numerous biomaterials have been developed over the years to enhance the outcomes of endoscopic sinus surgery (ESS) for patients with chronic rhinosinusitis. These products are specifically designed to prevent postoperative bleeding, optimize wound healing, and reduce inflammation. However, there is no singular material on the market that can be deemed the optimal material for the nasal pack. We systematically reviewed the available evidence to assess the functional biomaterial efficacy after ESS in prospective studies. The search was performed using predetermined inclusion and exclusion criteria, and 31 articles were identified in PubMed, Scopus, and Web of Science. The Cochrane risk-of-bias tool for randomized trials (RoB 2) was used to assess each study’s risk of bias. The studies were critically analyzed and categorized into types of biomaterial and functional properties, according to synthesis without meta-analysis (SWiM) guidelines. Despite the heterogeneity between studies, it was observed that chitosan, gelatin, hyaluronic acid, and starch-derived materials exhibit better endoscopic scores and significant potential for use in nasal packing. The published data support the idea that applying a nasal pack after ESS improves wound healing and patient-reported outcomes.

## 1. Introduction

Chronic rhinosinusitis (CRS) is a debilitating disease that affects 5% to 15% of the global population [1]. This disorder is characterized by prolonged inflammation of the nasal mucosa, which leads to olfactory dysfunction and other symptoms such as runny nose, congestion, itchiness in the nasal cavity, sneezing, loss of smell, and headache [1,2]. Indeed, these dysfunctions may result in reduced quality of life, failure to perceive danger, social anxiety, and psychological problems [3].

Current treatments for CRS and active sinus infection include corticosteroids, saline irrigation, antihistamines, and antibiotics [4,5]. However, in patients who do not respond to the first-line treatment choice, functional endoscopic sinus surgery (FESS) will be the following approach to alleviate the symptoms [1,5]. FESS or ESS is the standard surgical intervention that involves the excision of the nasal mucosa and bone to remove obstructions and open up the sinus ostia [6,7]. A successful FESS will result in accessible and open sinus ostia, thus re-establishing ventilation and drainage. Subsequently, follow-up medical therapy can be delivered effectively to the nasal cavity to reduce recurring mucosal inflammation. [7,8].

However, postsurgery complications, such as adhesion (synechiae) or ostial stenosis, are prone to occur due to the presence of blood clots and the nature of the denuded wounded surface in the nasal cavity itself [9,10,11]. Revision surgeries are required to remove the adhesions. Various techniques have been introduced to manage postsurgery complications, which include middle turbinate resection, “controlled synechiae” formation, middle turbinate suturing, and placement of absorbable or nonabsorbable packs [10].

For decades, nasal packing has been used to reduce the side effects of FESS, such as postoperative epistaxis, adhesion prevention, and wound healing acceleration [1,12]. In addition to stopping bleeding and synechiae from forming, the nasal pack also helps stabilizes the cartilaginous and bony framework of the nasal cavity [13]. Even though nasal packing does not seem to be important in nasal surgical intervention, it can have an impact on the entire healing process and may play a significant role in the surgical outcome.

Over the years, various nasal packing technologies, including gauze-based, sponge-based, and balloon-based systems, have been developed [9]. Nasal pack technology continues evolving due to several limitations which usually affect the patient, such as discomfort, difficulty in removal, and potential for infection. Several ingenious strategies have been implemented in an effort to surmount these limitations, including nanomaterial-based systems that provide targeted and sustained drug delivery, as well as enhanced wound healing [14,15]. These systems can be developed to have certain characteristics, such as controlled release kinetics and antimicrobial capabilities, and can be adapted to the need of each patient [16]. However, additional study is required to establish their safety and effectiveness before these technologies can be extensively employed in clinical practice, because they are still in the early phases of development.

**Figure 1:**
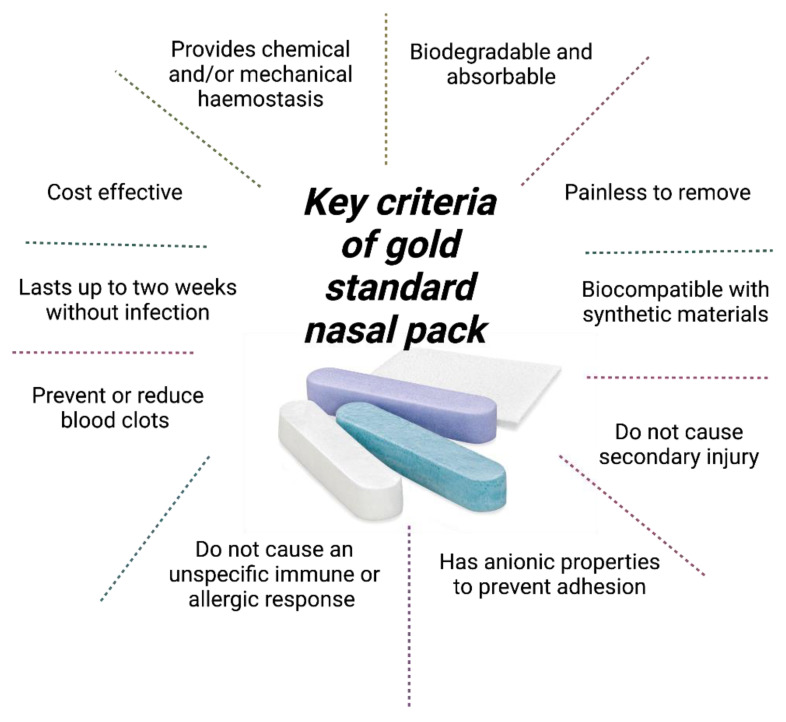
Key criteria of gold standard nasal pack.

According to Park et al., the nasal pack gold standard comprises the following criteria: (1) biodegradable and absorbable, (2) painless to remove or does not cause secondary injury to the wounded site, (3) provides chemical and/or mechanical hemostasis, (4) prevents/reduces blood clots, (5) lasts up to two weeks without infection, (6) has anionic properties to prevent adhesion, (7) does not cause an unspecific immune response or allergic reaction, (8) biocompatible with synthetic materials, and (9) cost-effective [17] (Figure 1).

Various synthetic and natural nasal packing is currently being researched, and some of the nasal pack is already available on the market. Chitosan [11,18,19], calcium alginate [17,20], starch [9,21], fibrin/fibrinogen [5,6,22], gelatin [23,24,25,26], polyvinyl acetate (PVA) [1,13,22,27], and silicone [10,28] are among the material used for nasal packing. Nasal pack comes in the form of gel, foam, sponge, or solid, with various shapes and sizes determined by the manufacturer. 

Functional biomaterials are materials designed to interact with the living system and perform intended functions within the body [29]. The concept of functional biomaterials can be utilized to develop the most advantageous nasal pack. Nasal pack functions include hemostasis, topical medication delivery, and adhesion prevention [30]. 

While various synthetic and natural materials, such as chitosan, calcium alginate, and gelatin, have been researched and are already in use for nasal packing, the heterogeneity of outcome measures makes it difficult to determine the best biomaterial in rhinology. 

It is worth noting that there is currently no single ideal material for nasal packing available on the market. Systematic research on biomaterials in rhinology, conducted in 2017 [31] and 2016 [30], revealed that the heterogeneity of outcome measures makes it challenging to identify the best biomaterial in rhinology. The systematic review searches included findings from the years 1990 to 2016, and only covered a range of materials, such as PVA, gelatin, gelatin–thrombin admixture, polyethylene terephthalate (PET)-coated cotton fleece, hyaluronic acid (HA), HA–collagen mixture, fibrin, cellulose, chitosan, carboxymethylcellulose (CMC), microporous polysaccharide hemospheres (MPH), polyurethane (PU), polylactide-co-glycolide (PLG), and polypropylene glycol (PEG) [30,31]. Considering the significance of evaluating the efficacy of present functional biomaterials for nasal packing, it is imperative to conduct a systematic review encompassing the most recent clinical trial data, spanning from 2014 up to the current time. Such a review would be beneficial in identifying the most advantageous material for nasal packing, and could potentially lead to improvements in patient outcomes and quality of life.

Therefore, this study aims to review the efficacy of current functional biomaterials for nasal packs through the data obtained from clinical trials starting from 2014 until recently. 

## 2. Materials and Methods

### 2.1. Review Question

The review search question was formulated using the ‘PICO’ framework, with patients undergoing ESS as the ‘Population (P)’ cohort, functional biomaterial as the ‘Intervention (I)’, and endoscopy score and patient-reported outcome as the ‘Outcome (O)’. No ‘Comparison (C)’ was defined. Hence, the formulated question was, “What are the most potential biomaterial that could improve endoscopy score and patient-reported outcome?”

### 2.2. Literature Search Strategy

This systematic review was reported according to the preferred reporting items for systematic reviews and meta-analysis (PRISMA) guidelines [32], with a comprehensive search of the relevant words or phrases in PubMed (National Centre for Biotechnology Information, NCBI, Bethesda, MD, USA), Scopus (Elsevier, Amsterdam, The Netherlands), and ISI Web of Science (WoS) (Clarivate Analytics, Philadephia, PA, USA). The search strategies used the following words or phrases: (anti-inflammat* OR antioxidant OR antibact* OR antimicrob* OR antivir* OR PH-responsive OR temperature-responsive OR thermoresponsive OR stimuli-responsive OR “chemical stimuli” OR “physical stimuli” OR “biochemical stimuli” OR “drug delivery” OR “drug eluting” OR incorporation OR “controlled release” OR “slow release” OR “controlled delivery” OR angiogenesis OR procoagulant OR hemocompatib* OR hemostasis OR mucoadhes* OR immunomodulation OR conjugat* OR biomimetic OR adjuvant OR bioactive) AND (biomaterial OR stent OR “nasal spacer” OR sponge OR gauze OR foam OR scaffold) AND (nasal OR rhinology OR sinonasal OR paranasal OR endonasal OR rhinosinusitis OR nasosinusitis OR fess OR “functional endoscopic sinus surgery”) NOT (brain OR colon OR pulmonary OR coronary OR carotid OR endocarditis OR spinal). 

Duplicate findings were sorted out using EndNote 20. The articles were screened by examining the title and abstract based on the inclusion and the exclusion criteria (refer to Section 2.3). After that, manual search was conducted by screening the references of the included studies to identify additional eligible studies. Further screening by reviewing the full text was performed according to the predefined exclusion and inclusion criteria.

### 2.3. Eligibility Criteria

Study selection was performed according to the eligibility criteria. The eligibility criteria include only those articles that were (1) written in English; (2) prospective studies; (3) published within a 10 year period; (4) underwent ESS as CR interventions; and (5) used biomaterials for packing after ESS procedure. Studies that were (1) in the forms of review articles, notes, letters, and book chapters; (2) published before 2014; (3) not written in English; (4) animal, in vitro, and bench studies, (5) retrospective, case studies, or cohort studies; and (6) not involving ESS procedures, were excluded from this review. Two reviewers worked independently in determining the eligibility of articles to be included or excluded in this study.

### 2.4. Data Extraction and Synthesis

From the selected articles, data were extracted from texts, tables, figures, and Supplementary Materials. The initial findings were arranged in a data extraction table, which includes (1) source; (2) type of biomaterials used in both the intervention and control; (3) outcome parameters; (4) measurement time point; and (5) biomaterial functional properties mentioned in the text or Supplementary Material. During data extraction, significant heterogeneity was observed in the type of biomaterials used, study methodologies (i.e., parameter and measurement time point), and in the data reporting systems. Therefore, formal meta-analysis of the variable cannot be accomplished, and a systematic review without meta-analysis (SWiM) was performed [33]. The PRISMA 2020 checklist and synthesis without meta-analysis (SWiM) reporting items are provided as the Supplementary Material. 

Next, in order to answer the review question, the obtained data were aggregated according to types of material, and the effect direction plot was tabulated, which consists of (1) intervention/control; (2) time point; (3) study size; (4) nasal endoscopy score; (5) patient-reported outcomes; (6) other parameters; and (7) references. The effect direction for the outcome domain for each study was then visually represented as an upward, downward, or bidirectional arrow. Upward arrow ▲ = positive score, downward arrow ▼ = negative score, sideways arrow ◀▶ = no change/mixed effects/conflicting findings [34]. The study quality was denoted by the reference column’s colour shading: green = high-quality/low risk of bias; yellow = moderate/some concerns; red = low-quality/high risk of bias. 

### 2.5. Risk of Bias

Two reviewers worked independently in determining the risk of bias of each article in this study. The Cochrane risk-of-bias tool for randomized trials (RoB 2) was used to assess each study’s risk of bias. This tool comprises a five-item checklist: (1) randomization process; (2) deviations from the intended interventions; (3) missing outcome data; (4) measurement of the outcome; and (5) selection of the reported result.

## 3. Results

### 3.1. Data Retrieval

**Figure 2:**
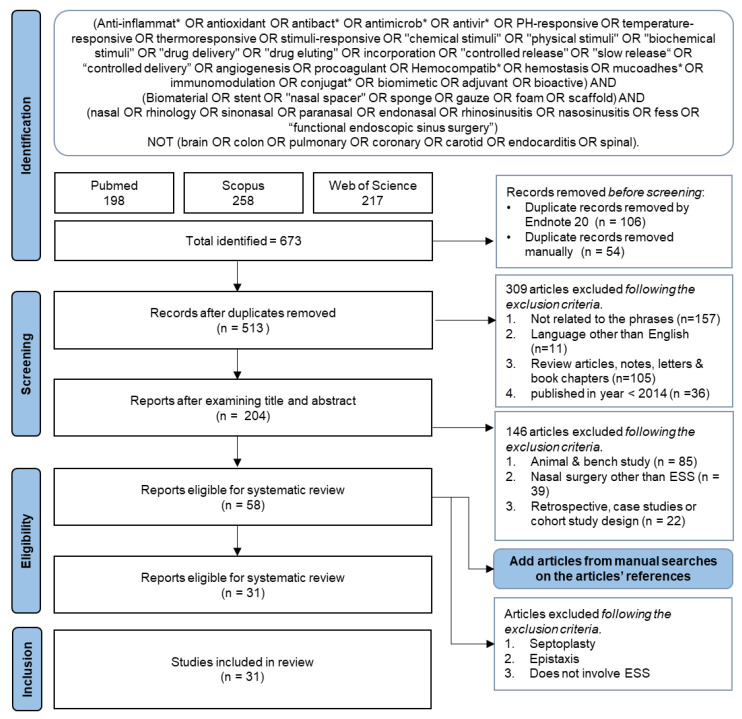
PRISMA flowchart summarizing the study screening and selection procedure. PubMed, Scopus, and Web of Science (WOS) were searched for relevant articles.

A comprehensive search of the relevant words or phrases in PubMed (National Centre for Biotechnology Information, NCBI, Bethesda, MD, USA), Scopus (Elsevier, Amsterdam, The Netherlands), and ISI Web of Science (WoS) (Clarivate Analytics, Philadelphia, PA, USA) retrieved a total of 673 articles. All articles were pooled into Endnote software v20, and 160 duplicates were removed. Based on the abstract and title, 309 articles were excluded for being unrelated to the search phrases and not written in the English language. In addition, review articles, case studies, letter and book chapters, and articles published before 2014 were also not included. After that, the remaining 204 articles were read in full to ensure the study was investigating the effect of biomaterials in rhinology. A total of 146 articles that were animal and bench studies, about nasal surgery not related to ESS, and of a retrospective or cohort study design were further excluded. Additionally, we performed manual searches on the articles’ references to ensure all related studies are included, which brings the total number of manuscripts to 58. A final screening was performed to ensure that only studies related to the usage of functional biomaterials during ESS are included in this review. Finally, 31 articles were chosen to be included in this systematic review. The flowchart in Figure 2 summarizes the study selection approach. 

### 3.2. General Characteristics of the Included Studies

**Figure 3:**
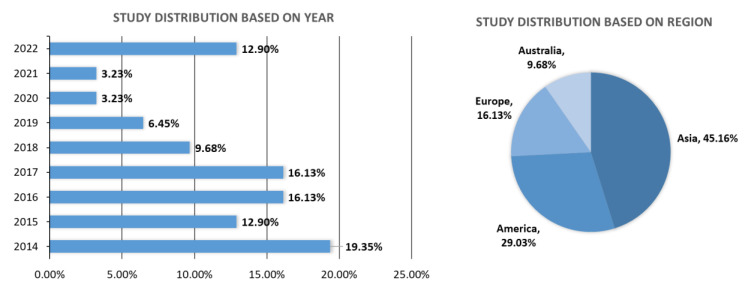
Study distribution based on year of publication and region where the study took place.

The 31 studies eligible to be included in this review were published between 2014 and October 2022 (Table 1). There was a decreasing trend of study distribution after 2014; however, in 2022, there was a significant increase in the publications on nasal pack material used after ESS. Fifteen countries contributed to the pools of publication, with 46.88% of it coming from Asia (China, India, Iran, Malaysia, Nepal, South Korea, and Vietnam) (Figure 3).

### 3.3. Types of Biomaterials Used as Nasal Pack

**Figure 4:**
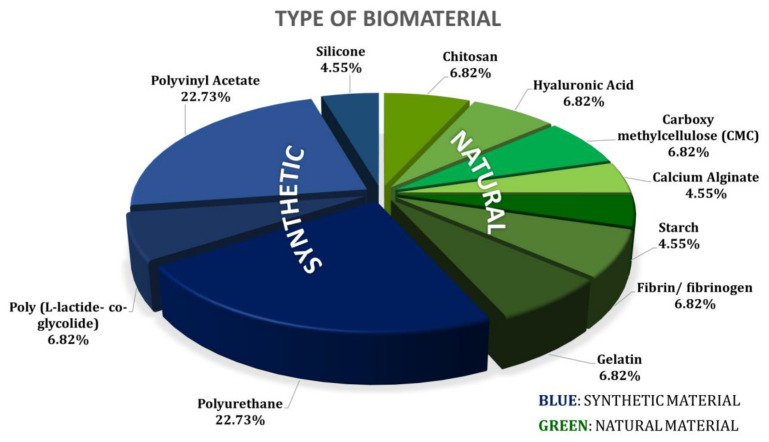
Types of biomaterials in nasal pack.

Based on the 31 articles that are included in this study, 12 types of biomaterials have been identified (Figure 4). Biomaterials made of polyvinyl acetate and polyurethane are the most studied materials in terms of nasal packing after ESS. Chitosan, hyaluronic acid, carboxymethylcellulose, calcium alginate, starch, fibrin/fibrinogen, Propel stent, gelatin, and silicone are among the materials that are studied in this topic of interest. 

### 3.4. Natural vs. Synthetic

For further analysis, the biomaterials that are mentioned in the included articles are divided according to the natural or synthetic nature of the material. Synthesis without meta-analysis (SWiM) was performed by plotting the effect direction of the outcomes.

#### 3.4.1. Natural Biomaterial

Several natural biomaterials, such as chitosan, carboxymethylcellulose (CMC), calcium alginate, fibrin/fibrinogen, gelatin, hyaluronic acid, and starch-derived material, have been identified and are tabulated in Table 2.

##### Chitosan

Three studies use chitosan-derived material. Chitosan nasal pack without any additive (Def, GaPP, or budesonide) showed improvement in the overall nasal endoscopy score and degree of crusting as early as 2 weeks [11,18]. The addition of budesonide further improved the scores of nasal endoscopy at all tested times [11]. At week 12, the difference in percentage between chitosan and control for incidence of adhesions, edema, and granulation tissue formation is larger than 48%; however, there were not many differences in incidence of pus and crust formation, with only 20% difference between chitosan and control [11]. This was supported by another study that shows that there is no improvement between chitosan and control in overall nasal endoscopy score at week 12 [19]. However, there were improvements in endoscopy score and images when comparing chitosan and control to the score and images from earlier time points (week 0 and 2) to week 12 [11,19]. Ostium patency was tested in two studies [11,19]. It was shown that chitosan-derived material increases the ostium patency score compared to the control group at all tested ostia (frontal, sphenoid, and maxillary) as early as week 2 [11,19]. Only one study reported input from the patient through SNOT-22 and VAS on week 12 [19]. It was shown that chitosan has better outcomes in SNOT-22 and the VAS scores for facial pain/discomfort, bleeding, nasal obstruction, anterior and postnasal secretions, and sense of smell, which improve significantly in patients receiving only chitosan nasal pack. The addition of both Def and GaPP increased patient outcomes, though it was not significant [19].

##### Carboxymethylcellulose (CMC)

CMC was used in three different studies. Overall, the total endoscopic score seems to be higher for CMC when compared to the other intervention (hyaluronic acid (HA)) [12]. Matheny et al. reported that 24 out of 29 patients achieved less than 25% synechiae formation at 6 and 12 weeks; however, lower synechiae formation was found in patients using HA as the intervention [12]. A study by Park et al. reported that the incidence of adhesion and edema for CMC vs. calcium alginate shows no difference between groups, though when looking at the severity of adhesion and edema, the group with CMC showed higher severity [17]. This is similar to the study by Antisdel, where synechiae formation was not significantly different between interventions. However, it was noted that there were increases in debridement requirement, amount of crusting, and granulation formation in the CMC group [9]. The patient-reported outcome (VAS) shows no significant differences for postoperative pain, discomfort from nasal discharge, or pain during packing removal between calcium alginate packings and carboxymethyl cellulose packings [9,17].

##### Calcium Alginate

Two research studies employ calcium alginate as nasal pack. A study by Hwang et al. shows that the usage of calcium alginate with steroid results in lower POSE scores compared to calcium alginate soaked with saline. Overall endoscopy scores, middle turbinate lateralization, polypoid degeneration of the ethmoid cavity, and sphenoid sinus severity were significantly lower in the group of patients using calcium alginate soaked with steroid as the intervention [20]. Park et al. shows the incidence of adhesions and edema with calcium alginate packing gradually decreased during the study period, and the severity of adhesions and edema was lower with calcium alginate than with carboxymethylcellulose at all time points (significant at week 4) [17]. Patient-reported outcome (VAS) shows no significant differences for postoperative pain, discomfort from nasal discharge, or pain during packing removal between calcium alginate packings and carboxymethylcellulose packings [17].

##### Fibrin/Fibrinogen

Three studies reported on the usage of fibrin-derived material, where two studies use fibrin sealant (FS) and one uses SinuBand. The results show that there was no significant difference in the mean endoscopic score between the side packed with FS and the side packed with Nasopore [6,22]. However, during postoperative endoscopic examinations, remnants of Nasopore packing material were observed in a higher number of patients and at a longer duration than FS-treated patients. The mean score for crusting at 1 and 2 weeks postop was also significantly lower in the FS-treated side than in the Nasopore- and Merocel-treated side [6,22]. Additionally, the Nasopore- and Merocel-treated side had more severe granulation formation than the FS-treated side at 1 month postsurgery [6,22]. There were no significant differences between the FS- and Nasopore-packed sides in terms of the mean scores for adhesion, bleeding, infection, or frontal sinus ostium stenosis during the follow-up period. The general satisfaction score for FS was slightly higher than Nasopore and Merocel, but the difference was not statistically significant [6,22]. The average VAS scores for the two sides (Merocel and Nasopore) showed no significant difference, except for nasal obstruction. Adriaensen et al. found that SinuBand FP resulted in significantly lower polyp scores compared to Merocel, and a lower percentage of sinuses developed mucosal polypoid change through the first 30 and 60 days postsurgery. However, there were no significant differences in inflammation, adhesion, or endoscopy scores between SinuBand FP, Merocel, and SinuBand [5].

##### Gelatin

There were four studies that reported on utilizing gelatin. The overall endoscopic score shows a significant reduction in value after one month of surgery and during the follow-up period [24,25]. During the 3rd and 6th weeks, steroid-loaded Gelfoam demonstrated a better endoscopy score compared to saline-loaded Gelfoam [25]. However, this finding was different from Sow, where they found out that Gelfoam with saline has better outcomes at 3 weeks postsurgery [26]. They also did POSE scoring, where the Gelfoam loaded with steroid shows better outcomes at one week and three months postsurgery [26]. However, each outcome was not significant. When comparing between different types of gelatin sponge, Cutanplast has better LKS, POSE, discharge, polyp, and edema scores compared to Spongostan at 2 weeks, 1 month, 2 months, and 3 months. No significant differences were found at 6 months [24]. VAS patient symptoms decreased toward day 30 postsurgery. When comparing between the Cutanplast and Spongostan packing, Cutanplast has better scores in terms of discomfort, postnasal drip, rhinorrhea, headache, and pain compared to Spongostan packing [24]. In terms of remaining material left inside of the nasal cavity, it was found that Cutanplast reduced faster compared to Spongostan.

##### Hyaluronic Acid

Three studies reported on the application of hyaluronic acid after ESS. The overall endoscopy score and synechiae formation show a statistically significant improvement at 6 and 12 weeks. However, when compared to the other interventions, there are no statistically significant differences in terms of edema, crusting, or mucopurulent [12]. Hyaluronic acid with the addition of steroid shows a significantly better endoscopic score and reduction in mucus secretion compared to the other group [40]. However, there were no significant differences between the group for edema, crust, scar, and polyposis [40]. A study by Long et al., comparing hyaluronic acid to Merocel, shows that a better LKES ratio was observed in the HA group, especially on day 2 until day 6 [27]. Patient-reported outcome shows improvement in quality of life based on SNOT-20 scores compared to previous SNOT-20 scores [12]. When compared to the Merocel group, VAS outcome was significantly higher [27].

##### Potato Starch-Derived Material

There were two research studies that reported on the application of starch-derived materials following ESS. Synechiae formation is not significantly different between the three group (CMC, potato starch MPH, and potato starch wafer); however, in terms of the severity, debridement requirement, crusting, and granulation, starch-derived material showed a better score than CMC [9]. When compared to Nasopore, the degree of crusting, remnant material, and need for debridement are better in material derived from starch [21]. Patient-reported outcome shows no statistically significant differences in pain, obstruction, bleeding, and discharge [9]. However, Schlewet et al. reported that there is a high incidence of infection in the group treated with starch-derived material compared to Nasopore [21].

#### 3.4.2. Synthetic Biomaterial

Several synthetic biomaterials, such as polyurethane, polyvinyl acetate, silicone, and poly(L-lactide-co-glycolide), have been identified and are tabulated in Table 3.

##### Polyurethane

Seven articles report on the comparison between Nasopore and other interventions such as modified amylopectin [21], fibrin sealant [6], chitosan nasal dressing [18], and Merocel (polyvinyl acetate) [36,37,38]. Three other articles report on the usage of Nasopore as a drug carrier for triamcinolone (TA) [3,8], oxytetracycline, and hydrocortisone [7], bringing the total included studies up to ten articles.

All seven studies show unfavourable effects on the endoscopy score when comparing Nasopore to their respective interventions. The sites tested with Nasopore show a higher degree of crusting [18], more retained material [6,18,21], more severe granulation [6], and a higher need for endoscopic debridement [6] than the intervention sites. Nonetheless, there is no significant difference between the intervention and Nasopore in terms of the mean score for adhesion [18,21], bleeding, or stenosis [6]. Nasopore has lower infection rates compared to the interventions [21]. In terms of patient-reported outcome, Nasopore shows no significant difference between interventions [6]. However, there were significant differences for nasal obstruction [6,7]. 

When comparing Nasopore with no packing, there were no differences in bleeding tendency, synechiae formation, mucosal edema, or crusting in the 1st week; however, starting in the 2nd week, there were significant improvements in the ostial patency. There were no significant differences in synechiae formation [7,35]. Subsequently, on week 12, OMC patency scores, synechiae formation, and nasal discharge were significantly better on the Nasopore side [7]. There was no significant difference between the two sides in terms of postoperative bleeding or nasal breathing. Interestingly, a study by Kastl et al. shows that patients reported feeling more pressure on the unpacked side on days 2 and 3 postsurgery [38].

Alternatively, when comparing Nasopore to the nonabsorbable Merocel, there was no statistical difference in the hemostatic property between the two materials in the immediate postoperative period [36]. However, patients with Merocel packs had better hemostasis until they were removed on the first postoperative day compared to those with Nasopore packs. Nasopore was found to be more mucosal-friendly, with a reduced incidence of adhesions, synechiae, and edema. No significant difference was observed in the incidence of infection between the two groups. Patients who received Nasopore had statistically significant benefits over those who received Merocel with regard to compliance and comfort levels. None of the patients showed willingness to re-use Merocel again, while 70% of patients were willing to try Nasopore in the future. Nasopore was not associated with pain on removal, unlike Merocel. However, another study that reported a long-term outcome of using Nasopore shows no statistically significant difference between the two types of packing with regard to disease outcome at 1 year postsurgery. The study also assessed secondary outcomes related to wound healing at various time points after surgery. The study found no statistically significant differences between the two types of packing regarding middle turbinate synechia, presence of crusts, appearance of secretions, mucosal edema, or presence of granulation tissue [37]. Additionally, there was no statistically significant difference between the two types of packing with regard to re-epithelialization at various time points after surgery.

Next, when comparing between drug-loaded Nasopore and the other interventions, endoscopy scores were significantly different at 1 and 2 months, with drug-loaded Nasopore giving positive effects [3]. Other than that, the Nasopore group also had better mucosal healing, with re-epithelialization reaching over 95.7% at day 30 compared to the control group [7]. No statistically significant difference in synechiae was found between Nasopore loaded with drug compared to the gauze strip loaded with drug. In terms of patient-reported outcome, no difference was observed between Nasopore loaded with drug and the other interventions [7,8,18], except for pressure [7].

##### Silicone

Two studies reported on the application of silicone. [28] Manji et al. show that the incidence of synechiae did not differ significantly between the Silastic stent and gloved-Merocel (GM) group, and at 3 months postsurgery, the incidence of synechiae was low in both groups. Pain reported during Silastic spacer removal was more severe than with GM spacers, but severity of other symptoms did not differ significantly between the two groups. There was no significant difference in endoscopic scores or likelihood of synechiae between the two groups at 5 weeks and 12 weeks postsurgery [28]. Another study by Chan et al., comparing a nonstent group and a Silastic stent group, shows that the stent side demonstrated a statistically significant decrease in adhesion formation at both 2 weeks and 8 weeks postsurgery. Similarly, crusting at week 2 postsurgery was reduced on the stent side, but this benefit resolved by the 8-week follow-up. At the 6-month follow-up, there was no change in the nasal endoscopy scores for patients compared to their assessment at 8 weeks [10].

##### Poly(L-lactide-co-glycolide) (PLG)

Two articles were reporting the usage of a PLG stent. The studies used Propel steroid-eluting stents vs. Merocel [1] and no stent [39]. The patient-reported outcome and middle lateralization scores are better on the Merocel side compared to the side with the PLG stent. However, there is no difference between Merocel and the PLG stent in terms of overall endoscopic score [1]. When comparing the PLG stent with no stent, the total MLM score, CRS-PRO, SNOT-22, and endoscopic severity scores were similar between the two treatments. However, the frontal MLM decreased in the MES group to a level below that of the non-MES group. Both groups showed significantly improved total MLM [39].

##### Polyvinyl Acetate (PVA) 

Ten articles reported the comparison between Merocel and other interventions such as hyaluronic acid [27], hemostatic gauze [13], PLG stent [1], silicone [28], fibrinogen [5], fibrin [22], and polyurethane [36,37]; two studies compared drug-eluting PVA with saline-soaked PVA [2,41]. 

There were some differences in the overall endoscopy scores. Merocel has been shown to be inferior to hyaluronic acid [27] and Nasopore [36]. According to the findings, patients who used a biodegradable gel of hyaluronic acid and berberine hydrochloride during surgery had better nasal patency than with Merocel [27]. When compared to the absorbable nasal pack, Nasopore, it is proven to be significantly more mucosal-friendly, with a reduced incidence of adhesions, synechiae and edema [36]. On the other hand, there is no difference between Merocel and the other interventions in terms of the endoscopic score when compared to silicone [28], fibrinogen/fibrin [5,22], PLG stent [1], and Nasopore [37]. One study shows that Merocel performed better than the PLG stent in terms of middle turbinate laterization [1].

The patient-reported outcome shows inferior results for Merocel when compared to hyaluronic acid [27], PLG coated with hemostatic gauze [13], and fibrinogen/fibrin [5,22], due to most patients feeling pain upon removal. 

#### 3.4.3. Functional Biomaterial

**Figure 5:**
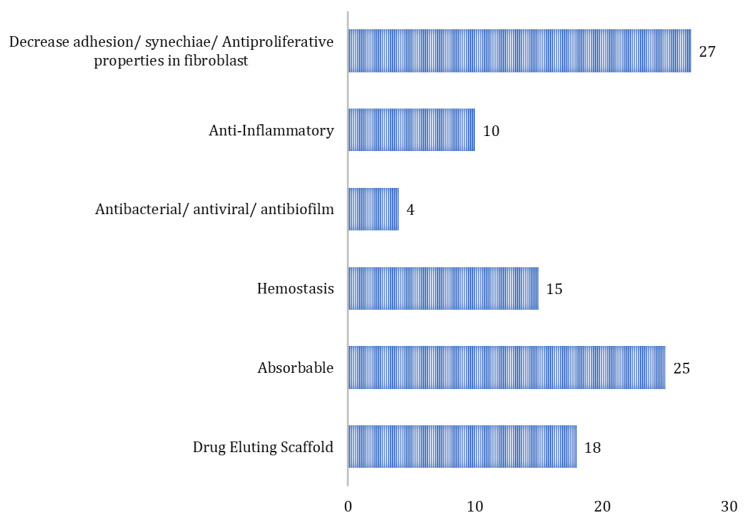
Six primary functions have been identified: drug-eluting, absorbable, hemostasis, antibacterial/antiviral/antibiofilm, anti-inflammatory, and reduce adhesion/synechiae.

Included articles were also categorized according to the function that the biomaterial may exert; six primary functions have been identified (Figure 5). There are 28 articles reporting that the tested biomaterials help to decrease adhesion and synechiae. Meanwhile, only four are reported to be antibacterial/antiviral/antibiofilm.

### 3.5. Risk of Bias

**Figure 6:**
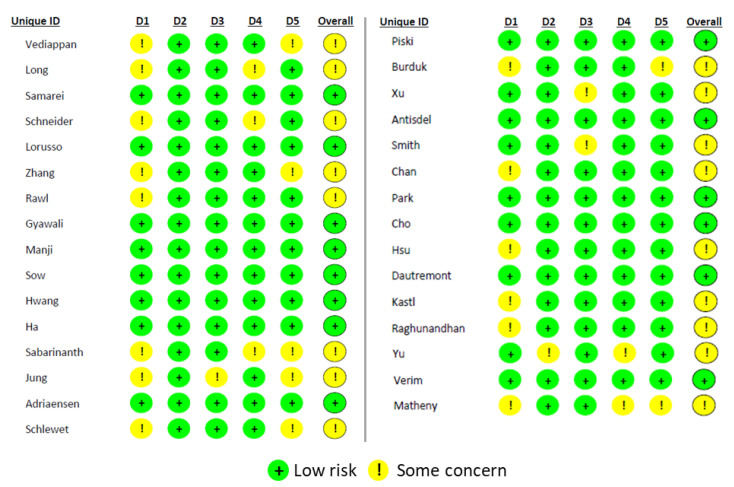
Risk of bias assessment of 31 reviewed articles according to RoB 2 tools. (D1) Randomization process; (D2) deviations from the intended interventions; (D3) missing outcome data; (D4) measurement of the outcome; and (D5) selection of the reported result.

In this review, all 31 articles were subjected to risk of bias analysis; 14 articles have low risk, and 27 articles are tagged with some concern (Figure 6). None of the studies had any unreliable data. 

## 4. Discussion

Restoring normal nasal airway anatomy and its function is one of the key goals following sinus surgery. To assess the success of the surgery, the surgeon will examine various postoperative outcomes, such as ostial patency, edema, discharge, crusting, bleeding, and the presence of synechiae. These factors are crucial in determining the effectiveness of the surgery and the overall healing process of the patient.

Ongoing bleeding and formation of synechiae are the two most common complications following ESS [9,11]. Synechiae occurs when two opposing denuded mucosal surfaces come into contact with each other following sinus surgery [43,44]. These scar tissues form between the nasal septum and the turbinate, and will obstruct the flow of air and mucus. The incidence and the severity of synechiae depends on the inter-relationships between fibrinolysis and fibroblast-initiated collagen deposition, which can be influenced by clot formation, granulation tissue formation, crusting, and fibrinous exudate secreted at the wounded surgical site [9,17,45,46].

A number of intraoperative and postoperative approaches have been developed to reduce the occurrence of stenosis and adhesions. Middle turbinate resection [47], or “controlled synechiae” formation or the Bolgerization technique [1,48], are intraoperative techniques to reduce adhesion. Postoperative approaches, such as adequate nasal irrigation, applications of topical nasal steroids, or regular endoscopic debridement, are generally preferred by surgeons in reducing adhesion [11]. However, none of the techniques have been deemed the gold standard in preventing synechiae. 

Another technique to reduce synechiae is by utilizing physical barriers such as nasal pack, spacer, or stent after surgery. Nasal packing is a practice often utilized after sinus surgery and septoplasty. The application of nasal pack prevents bleeding, stops synechiae from forming, and maintains the nasal structures, hence reducing postoperative problems. The idea of separating two denuded mucosa using biomaterials has been in practice for more than 3 decades [1,37,44]. There is a wide range of biomaterials currently available, though the search for the optimal nasal pack biomaterial is still ongoing. 

This review summarized the functional biomaterials with the greatest potential to be utilized as nasal packs. Due to the heterogeneity of the study designs and the articles’ contents, synthesis without meta-analysis (SWiM) was used to analyze the data [33]. There are studies published before 2014, which have been reviewed by Massey et al. [30,31], focusing on biomaterials in rhinology and absorbable pack. In this investigation, we therefore focused on the most recent findings regarding the implementation of functional biomaterials in ESS patients. Thirty-one articles published between 2014 and October 2022 were identified. After 2014, the number of studies on nasal pack application declined, with the lowest numbers occurring in 2019, 2020, and 2021. This phenomenon may be caused by the COVID-19 pandemic, which caused a temporary shutdown. The articles were also evaluated and classified according to biomaterial categories and their functions. There are 12 categories of biomaterials identified.

On the basis of the regions that contributed to the publication pools of using nasal pack as the intervention following endoscopic nasal surgery, it was also observed that the number of Western studies (54.84%) is comparable to Asian studies (45.16%). According to a recent review by Chee et al. (2022), there may be genetic and environmental distinctions between Western and Asian populations that influence the onset and progression of chronic rhinosinusitis (CRS) [49]. Nonetheless, the review concluded that additional research is necessary because CRS in Asia may be identical to CRS in the Western countries, and should be considered a global disease. 

### 4.1. Type of Biomaterials

#### 4.1.1. Natural Biomaterials

##### Chitosan

Chitosan is a polysaccharide that is derived from chitin, which is a natural polymer found in the exoskeletons of crustaceans such as shrimp, crab, and lobster, as well as in the cell walls of fungi [18]. Chitosan has several remarkable properties that make it suitable to be used in biomedical applications. It is a hemostatic agent [50,51], has mucoadhesion properties when in contact with mucin [52,53], has antimicrobial properties [54], provides an analgesic effect [55], and is biodegradable with nontoxic by-product [56]. Additionally, chitosan is also reported to aid in wound healing acceleration [52].

Based on the current review findings, there are two types of chitosan nasal pack used by Vediappan et al., Ha et al., and Hsu et al., which are Chitogel™ or CD gel [11,19], and Posi-Sep^®^ X [18]. Chitogel or CD gel is made of chitosan and dextran aldehyde, while Posi-Sep is made of N,O-carboxymethyl chitosan. N,O-carboxymethyl chitosan (NOCC) is a chitosan derivative in which carboxymethyl groups are added to the nitrogen and oxygen centres of chitosan to generate a water-soluble, negatively charged, biocompatible polymer that is hydrophilic, lubricious, and viscoelastic [57]. Generally, chitosan biomaterials improve the overall endoscopy score and degree of crusting as early as week 2 for all three studies [11,18,19], and the incidence of adhesion is low compared to control [11]. The mechanisms of Chitogel or CD gel in preventing adhesion are not completely understood. It is believed that the chitosan component might provide the hemostatic action, while the dextran component inhibits fibroblast migration and proliferation, allowing re-epithelialization and reciliation to occur before adhesion takes place [58,59]. 

The surgical outcomes continue to improve until week 12, though there were no differences between the intervention and control in terms of overall endoscopy score, which is probably due to the wound site already being healed [60]. The first two weeks following surgery are critical for wounds to heal and epithelization to occur; monitoring during this time is crucial, as it was observed that material retained in the nasal cavity might cause problems with wound healing and patient comfort [17,18,60].

##### Carboxymethylcellulose (CMC)

Carboxymethylcellulose is an anionic polysaccharide made from cellulose that transforms into a hydrocolloid when interacting with sodium chloride [17,61]. In the current market, CMC is available in the form of an injectable viscous material and mesh, which provides a physical barrier that suppresses adhesion formation by supporting and separating the damaged mucosal tissue. 

When comparing CMC material to other interventions, the included studies demonstrate a detrimental effect on the overall endoscopic score and adhesion score. [9,12,17]. Additionally, in blinded studies, physicians noted that CMC gel does not easily clear through irrigations, and has a higher score in crusting, granulation, and need for debridement [9]. However, in the study by Park et al., which utilized CMC packing in the form of mesh, it was observed that CMC packing dislodged from the packing site as early as week 1 [17]. Thus, the form and size of the biomaterial may also have an impact on how adhesion, granulation, and crusting develop. The mechanisms behind these findings are yet to be understood; however, CMC is a hydrophilic biomaterial that, when inserted into the nasal cavity after surgery, can become saturated with blood and exudate, causing it to swell and adhere to the denuded mucosa [61]. 

##### Calcium Alginate

Calcium alginate is a gelatinous polysaccharide composed of calcium ions and alginic acid extracted from seaweed [62]. Calcium alginate absorbs excess wound exudate, which forms a hydrophilic gel that provides a moist wound healing environment [63]. Both included studies used Algi-Pack and tested for 1, 4, and 8 weeks [17,20]. In a study by Park et al., it was shown that Algi-Pack showed a superior effect compared to CMC-based material; significant low adhesion and edema scores were observed at week 4 [17,20]. Calcium alginate is a well-known nonadherent dressing, which explains the low adhesion score compared to CMC [64,65]. Calcium alginate also aids in the clotting mechanism, as it has a high calcium content that, when coming into contact with wound exudates containing sodium ions and ion exchange occurs, transforms insoluble calcium alginate into soluble sodium alginate. The release of calcium can be beneficial, as calcium ions that are released into the wound will aid in the clotting cascade [63,66].

##### Fibrin-Based Material

Two types of fibrin/fibrinogen materials are reported in the study. The first one is fibrin sealant, and the second one is SinuBand FP. Fibrin sealant (FS), or fibrin glue, is a surgical hemostatic/sealant/adhesive material that is biocompatible and biodegradable. It consists of human fibrinogen, human thrombin, and added components, such as human factor XIII and bovine aprotinin, which mimic the final steps of the physiological coagulation cascade to form fibrin clots. SinuBand is a sterile thin film that acts as a lining to separate opposing mucosa [67]. The structural properties of SinuBand derive predominantly from fibrinogen, which provides adherence to mucosal surfaces, as well as bioabsorbability. A fluticasone propionate-eluting version of SinuBand, SinuBand FP, contains 80 g of FP.

Fibrinogen is a soluble protein that circulates in the blood plasma. When a blood vessel is damaged, fibrinogen is converted into fibrin through a series of enzymatic reactions triggered by the activation of other clotting factors. Fibrin molecules then assemble to form a mesh-like structure, which is the basis of a blood clot. This fibrin clot traps platelets and red blood cells, forming a stable clot that helps to seal the damaged blood vessel and prevent further bleeding [68]. Due to its involvement in clotting mechanisms, it was believed that fibrin/fibrinogen-based materials will cause more crusting and lead to higher adhesion rates [22]. However, contrary to popular belief, the findings from these three articles show that fibrin-derived materials have lower crusting compared to the other interventions. This is probably because the fibrin-based materials used in the three studies started to degrade as fast as day 1. However, materials that are easily washed away or dislodged might increase the rates of adhesion [17], which explains why there is no significant difference between the intervention group and the control group.

There are several advantages in fibrin sealant applications; FS is effective in controlling bleeding in all wounds following FESS. By administering it via aerosol spraying, it can reach all the resected sinuses that cannot be accessed with nasal pack application. Additionally, FS does not disrupt nasal ventilation because it forms a thin layer. On the other hand, nasal packing may interfere with nasal breathing immediately after FESS, as the material’s volume can obstruct the nasal cavity until it begins to degrade. Another benefit of using SinuBand is that the application of SinuBand FP creates a steroid-eluting and adhesion-preventing lining in the surgical cavity, allowing for uninterrupted sinus drainage during the healing process. However, due the adhesive nature of fibrin, it has been associated with an increased risk of adhesion in the middle meatus. These drawbacks can be avoided by preventing trauma to the middle turbinates [6,22]. 

##### Gelatin

In the current study, three types of gelatin-based biomaterials have been used, which are Gelfoam, Cutanplast, and Spongostan. Gelatin is a by-product of natural collagen partial hydrolysis. Due to its beneficial characteristics, including low cytotoxicity and nonimmunogenicity, it is utilized as a natural material and is referred to as “generally recognised as safe” (GRAS) material by the FDA [69].

There are a few types of gelatin nasal pack available in the market, such as Gelfoam, Surgispon, Cutanplast, and Spongostan. Gelatin nasal pack is an absorbable, water-insoluble, porous, and pliable sponge, usually created from porcine gelatin. Different manufacturers might have different methods of manufacturing the gelatin, such as using different concentrations of gelatin, which leads to different porosity and pore size [24]. According to Cho et al., Cutanplast has more porosity, smaller pore size, and is softer and less adhesive compared to Spongostan, which explains why Cutanplast degrades faster, and which reduces the chance of granulation tissue ingrowth [24]. However, similar to other natural bioabsorbable material, gelatin may not provide enough support or pressure to effectively control bleeding.

##### Hyaluronic Acid

Hyaluronic acid has gained popularity in recent years due to its numerous benefits, particularly in the field of medicine. One such application is the use of hyaluronic acid as a nasal pack. Hyaluronic acid is a glycosaminoglycan containing no sulfate bonds that can be found in the extracellular matrix, especially in the connective tissue, skin, and joints [70]. It is a unique hygroscopic, viscoelastic, and mucoadhesive versatile material that has broad applications in the medical field.

##### Starch

Two types of starch-derived material have been used in the studies, which are (1) natural plant-based polysaccharides (modified amylopectin + hydroxyethylcellulose), and (2) potato starch-derived mucopolysaccharide hemospheres (MPH). The plant-based nasal dressing is made from purified potato starch that has been modified to contain amylopectin and hydroxyethylcellulose. This product is biodegradable, has been successfully used in other surgical settings, and has been shown to reduce bleeding and aid in adhesion prevention. Microporous polysaccharide hemospheres (MPHs), which are local hemostatic agents produced as a result of advanced bioengineering from purified potato starch, are one of the hemostatic agents used during surgery [71]. MPHs use a sophisticated polymer crosslinking process based on plants to produce ultrahydrophilic, biocompatible particles. By absorbing the liquid portion of the blood and increasing the concentration of platelets and coagulation factors, they aid in the coagulation cascade [72]. Regardless of the patient’s coagulation status, the clotting process begins upon contact. On uneven terrain and in challenging to access places, MPHs offer extensive area coverage. MPHs are indicated as an adjunctive hemostatic device in surgical procedures, with the exception of neurologic and ophthalmic procedures, to help control capillary, venous, and arteriolar bleeding when pressure, ligature, and other traditional procedures are ineffective or impractical [71].

#### 4.1.2. Synthetic Biomaterials

##### Poly(L-lactide-co-glycolide) (PLG)

Poly(L-lactide-co-glycolide) (PLG) is a biodegradable and biocompatible copolymer that has been widely used in the field of drug delivery and tissue engineering. It is a synthetic polymer that can be tailored to have a wide range of properties, such as degradation rate, mechanical strength, and hydrophilicity, by adjusting the ratio of lactide and glycolide monomers used in its synthesis [73]. The PLG nasal stent is designed to help keep the nasal airway open by providing structural support to the tissues inside the nose. The Propel stent is made of a biocompatible polymer PLG that gradually releases a corticosteroid medication over time. The medication is designed to reduce inflammation in the sinus tissue and prevent the formation of scar tissue, which can lead to blockages [1]. The Propel stent shape and action medialize the middle turbinate, and delivers a continuous dose of mometasone furoate. 

##### Polyurethane

Nasopore is a type of nasal packing material used to control bleeding and promote healing following nasal surgery. It is made of a biocompatible polyurethane foam that expands when it comes into contact with moisture, conforming to the shape of the nasal cavity and providing gentle pressure to the wound site. The foam is designed to gradually dissolve by fragmentation, eliminating the need for removal. Nasopore has been shown to be an effective and well-tolerated option for nasal packing in various types of nasal surgery, including septoplasty, turbinate surgery, and endoscopic sinus surgery. Nasopore, when compared to natural materials, is slightly inferior compared to natural materials, such as modified amylopectin [18], fibrin sealant [6], and chitosan nasal dressing [15]. However, due to its nature of expanding when wet, it can help in preventing adhesion and ostium patency. However, in terms of synechiae formation, there were no significant differences between the pack and no pack site. This is probably due to the way Nasopore degrades. As Nasopore degrades, it fragments into smaller pieces, which can be flushed out of the nasal cavity with irrigation. However, higher degrees of crusting and more material being retained can occur if irrigation is not performed properly.

##### Polyvinyl Acetate

Merocel nasal packs are a medical device commonly used for the control of nasal bleeding. They are made of compressed polyvinyl acetate (PVA) foam that quickly expands when inserted into the nasal cavity, applying pressure to the bleeding area and helping to control the bleeding. Merocel nasal packs are highly absorbent, but they are not absorbed by the body. Instead, they are designed to be removed after a few days, which is usually determined by the attending health professional. They are often used in nasal surgeries, sinus surgeries, and other interventions that may cause nasal bleeding. Merocel requires activation with saline, which causes the Merocel to swell and provide pressure to the wounded surface. However, it adheres to the incision site, the bleeding site, and other raw areas over the septum. The pack traumatises the nasal mucosa as it is being removed, altering mucociliary clearance, causing bleeding, increased crusting, inflammation, and the development of synechiae [74,75]. It was also noted that pain upon removal was reported as high for Merocel. Therefore, some modifications have been applied to ease this disadvantage, such as using finger gloved-Merocel [1]. 

##### Silicone

There are two articles that used a silicone stent, Silastic™, as the intervention after ESS. Silastic™ stent is a small, flexible, tube-shaped device made from silicone that is used to maintain the patency of a nasal passage [76,77], and has the properties of biocompatible, flexible, and effective in maintaining the patency of the nasal cavity. When undergoing nasal surgery, a Silastic middle meatal spacer is inserted into the nasal cavity to keep the nasal passages open, and to avoid the development of scar tissue after the procedure. The study reported by Manji shows that the incidence of synechiae did not differ significantly between the Silastic stent and gloved-Merocel (GM) group, and at 3 months postsurgery, the incidence of synechiae was low in both groups. However, Chan et al. reported that, when comparing nonstent groups and silastic stent groups, the nonstent side demonstrated a statistically significant increase in adhesion formation at both 2 weeks and 8 weeks postsurgery. Similarly, crusting at postoperative week 2 was found to be reduced on the stent side, but this benefit resolved by the 8-week follow-up [10]. The silicone stent is nonabsorbable and, therefore, it is able to maintain the patency and separation between two denuded surfaces after surgery. However, according to the patient, silicone stent removal was painful compared to the gloved-Merocel spacer [28]. However, as with any medical intervention, the benefits and risks of using a Silastic nasal stent should be considered on a case-by-case basis, taking into account the patient’s individual needs and medical history. Additionally, the effectiveness of Silastic stents may vary depending on the surgical technique, patient anatomy, and other factors [10,28].

### 4.2. The Functionality of Biomaterials Used as Nasal Pack

#### 4.2.1. Drug-Eluting Stent

The drug-eluting stent is one of the well-established strategies in managing symptoms after ESS, such as recurrent polyps, adhesion, and inflammation. Topical agents such as saline, corticosteroid, antibiotics, antifungals, and decongestants are generally used in treating rhinology problems [16].

Oral steroids are often prescribed to manage acute sinusitis symptoms; however, they are not advisable for long-term usage due their systemic adverse effects, which include metabolic disturbances, behavioural instability, and detrimental effects on bone health [78]. Therefore, topical administrations are preferable for their simplicity of administration and tolerability. However, the complex nasal anatomical structure might be challenging to apply topical interventions. For these reasons, much work has been dedicated towards the invention of a drug-eluting biomaterial that can be introduced post-ESS.

Several studies included in this review have incorporated drugs into the biomaterials, such as chitosan [11,19], calcium alginate [17], fibrinogen [5], gelatin [25,26], hyaluronic acid [27,40], polyurethane [3,7,8], PVA [2,41], and PLG [1,39]. The drugs that have been used are deferiprone (Def) and gallium protoporphyrin (GaPP) [19], budesonide [11,40], triamcinolone [2,3,8,20,25,41], fluticasone propionate [5], hydrocortisone [26], berberin [27], oxytetracycline and hydrocortisone [7], and mometasone [1,39]. It was shown that the aforementioned materials are able to be eluted with selected drugs, and the selected drugs were successfully delivered to the wound site. This can be seen by the resulting better performance in both the endoscopic score and patient-reported outcome in the site that received the drug-eluting stent [3,11,25,27,40]. 

#### 4.2.2. Absorbable vs. Nonabsorbable

The use of absorbable and nonabsorbable nasal pack is a common practice in managing postoperative bleeding and edema after endoscopic sinus surgery (ESS). Absorbable nasal pack is made of materials that degrade over time and do not require removal, while nonabsorbable nasal pack is made of materials that need to be removed after a certain period of time. There is still debate over which type is better, with some studies suggesting that absorbable nasal pack is more comfortable for patients and has fewer complications, while others suggest that nonabsorbable nasal pack is better at controlling bleeding and edema [17,18].

Nonabsorbable nasal pack requires removal, and the duration of the packing on site depends upon the physician, patient comorbidities, and bleeding severity. Usually, nonabsorbable packing, such as Merocel, can be removed 24–48 h after operation [79]. However, removing it may cause nasal tissue trauma and secondary injury. As a result, the patient reported that the removal was painful, and the prospect of the pack being removed caused anxiety. It was also reported that it may have a higher risk of complications, such as infection or mucosal damage, compared to absorbable nasal packs [22]. Nonetheless, nonabsorbable nasal pack provides great pressure to the wounded area and aids in homeostasis.

Recent developments on nasal pack have given rise to absorbable nasal pack. Several biomaterials have been used for absorbable nasal pack, such as chitosan [11,18,19], CMC [9,12,17], calcium alginate [17,20], fibrin/fibrinogen [5,6,22], gelatin [24,25,26], hyaluronic acid [12,27,40], starch [9,21], polyurethane [3,6,7,8,18,21,35,36,38], and PLG [1,39]. Endoscopic scores show that chitosan, gelatin, and hyaluronic acid have better performance in terms of synechiae formation, crusting, adhesion, and edema. Nonetheless, it has been observed that certain biomaterials, including PLG, polyurethane, calcium alginate, and fibrin/fibrinogen, exhibit similar endoscopic scores. Variations in these scores must be considered in the light of factors such as the manufacturing process, production parameters such as ingredient concentration, and the manner in which the material degrades (i.e., dispersal or fragmentation). 

#### 4.2.3. Hemostasis

The use of a nasal pack can aid in achieving hemostasis by several means, including applying pressure to assist with vasoconstriction, acting as a hemostatic agent itself, or releasing drugs with hemostatic properties.

Wound healing is a complex process that involves four overlapping phases: hemostasis, inflammation, proliferation, and remodeling [80]. Hemostasis occurs immediately after injury and involves blood clotting to stop bleeding. Inflammation begins soon after, and involves immune cells moving to the site of injury to fight infection and clear away debris. Proliferation involves the growth of new tissue to replace the damaged tissue. Finally, remodeling involves the reorganization and strengthening of the new tissue to improve its function and appearance [81].

Nasal pack such as PVA, which expands immediately upon contact with moisture (saline or liquid), would be an excellent candidate for assisting hemostasis. This expansion can help to apply pressure to the nasal mucosa, which can help to stop bleeding, and can also absorb excess blood and other fluids to further reduce bleeding. Surgical hemostatic/sealant/adhesive material that is biocompatible and biodegradable can also be used to assist hemostasis. However, different opinions exist, as the adhesive properties of nasal pack may increase the risk of adhesions in the middle meatus after FESS [22]. However, the findings from this review did not support the statement. 

Chitosan, hyaluronic acid, carboxymethylcellulose, calcium alginate, fibrin/fibrinogen, PVA, PLG, gelatin, and silicone have been reported to assist in hemostasis and wound healing. Another thing that needs to be taken into consideration is that nasal pack should not absorb moisture from the wound bed, as it will hinder wound healing [82]. Silicone has been found to aid in the healing of wounds by creating a protective barrier that can guard against infection and prevent the wound from becoming dehydrated [28,77]. Additionally, it can help minimize scarring by encouraging the development of a smooth and uniform surface at the wound site.

#### 4.2.4. Prevention of Adhesion and Synechiae Formation

The occurrence of nasal synechiae typically takes place during the healing process of the nasal mucosa, specifically during the proliferation and remodeling phases of wound healing [83]. Specifically, it happens when the injured surfaces of the nasal mucosa stick together, leading to adhesions or scars.

On the other hand, absorbable nasal packs may degrade too quickly or too slowly, leading to inadequate or excessive packing, which can also cause adhesions [24]. Additionally, the use of certain materials may trigger an immune response, leading to inflammation and scarring. Therefore, the choice of nasal pack material should be carefully considered to minimize the risk of synechiae.

Chitosan and hyaluronic acid are two materials that have been shown to be effective in preventing synechiae formation. Chitosan has been reported to promote tissue regeneration and prevent adhesion formation by reducing inflammation and promoting angiogenesis [11]. Hyaluronic acid has also been shown to prevent adhesion formation by providing a barrier to the wound site and promoting tissue regeneration [27].

#### 4.2.5. Inflammation

Chitosan, hyaluronic acid, and carboxymethylcellulose are known to possess anti-inflammatory properties. Chitosan can inhibit the production of inflammatory cytokines and reduce the infiltration of inflammatory cells to exert its anti-inflammatory effects [84]. Similarly, hyaluronic acid can suppress the activity of immune cells and decrease the production of inflammatory mediators to reduce inflammation [85]. In contrast, calcium alginate, starch, fibrin/fibrinogen, PVA, PLG, polyurethane, gelatin, and silicone do not have intrinsic anti-inflammatory properties, though they can indirectly contribute to reducing inflammation by promoting wound healing and tissue repair.

#### 4.2.6. Antibiotic, Antiviral

Chitosan and calcium alginate are the two materials found to have antimicrobial activity. Studies have demonstrated that chitosan possesses a wide range of antimicrobial properties, effectively combatting numerous types of microorganisms, such as bacteria, fungi, and viruses. Its mechanism of action involves disturbing the cell walls of microorganisms and hindering their metabolism, ultimately resulting in their demise [54]. Calcium alginate, on the other hand, has been found to have antibacterial properties, particularly against Gram-negative bacteria [86]. Calcium alginate is thought to operate by releasing calcium ions, which can interfere with the cell walls of bacteria, hindering their growth [87]. It should be emphasized that even though chitosan and calcium alginate exhibit antimicrobial properties, their efficacy may fluctuate depending on the microorganism being targeted, the concentration of the material employed, and other related factors.

### 4.3. What Is the Most Potential Biomaterial That Could Improve Endoscopy Score and Patient-Reported Outcome and Subsequently Improve Wound Healing after ESS?

Wound healing is a complex process that involves multiple stages and is influenced by various factors. The duration of each stage varies depending on the severity and type of wound, as well as individual factors such as age, health status, and underlying medical conditions. While a nasal pack’s primary function is to control bleeding, it is also essential that the nasal pack aids in the wound healing process. In this regard, the ideal nasal pack should possess all the necessary features that promote wound healing.

The literature indicate that an ideal nasal pack should possess several critical features, such as biodegradability, minimal pain upon removal, and providing hemostasis. Additionally, it should prevent blood clots, remain effective for up to two weeks without causing infection, and have anionic properties to deter adhesion. It should not elicit any nonspecific immune response or allergic reactions, be biocompatible with synthetic materials, and be cost-effective [10,12,17,37].

It is not feasible to determine which material is superior to others, since there are limited studies available from which to draw conclusive results due to the heterogeneity of the research. However, it has been observed that chitosan, gelatin, hyaluronic acid, and potato starch-derived materials have better endoscopic scores, and have the potential to be used for nasal packing. These natural materials can be optimized by adjusting the concentration and formulation to cater to the needs of individual patients. Each material possesses unique properties that make them suitable for different applications. 

One critical aspect of wound healing is to prevent synechiae or adhesions between the nasal mucosa and the septum. The material used for nasal packing should not promote fibroblast proliferation, which is a key factor in the development of synechiae. For instance, chitosan–dextran, a biomaterial with both chitosan and dextran components, has been shown to reduce fibroblast proliferation due to dextran’s ability to inhibit fibroblast migration and proliferation [11]. Additionally, the application of a steroid-eluting stent has also been shown to be effective in preventing synechiae.

Nasal packs such as PVA have been shown to be effective in controlling bleeding by swelling and applying pressure to the wound surface. The condition, shape, and size of the biomaterials used in nasal packing also play a critical role in promoting wound healing. The complexity of the nasal structure makes certain anatomical regions difficult to access, which makes aerosolized or gel-like materials more preferable.

Nonetheless, the findings of this review have significant clinical implications regarding the utilization of nasal packs in postoperative patients. The conventional nasal packs have been linked to notable patient discomfort and complications, including mucosal damage, bleeding, and septal perforation. Based on this review’s findings, the use of nasal dressings made from natural materials, such as chitosan and CMC, may offer a safe and effective alternative to traditional nasal pack. These dressings were found to be highly absorbent and effective at controlling bleeding, while also demonstrating excellent biocompatibility and promoting rapid healing. As such, they may be a valuable addition to the armamentarium of postoperative nasal care, allowing for improved patient comfort and reduced risk of complications. However, further studies are needed to fully explore the clinical potential of these dressings and to optimize their use in various clinical settings.

In summary, the ideal nasal pack should not only control bleeding, but also promote wound healing by possessing the necessary features and conditions. Factors such as the shape, size, and material used in the nasal pack should be carefully considered to ensure that the pack aids in the healing process and prevents complications such as synechiae. 

### 4.4. Limitations of the Study

The present study has certain limitations that warrant careful consideration when interpreting the results. One significant limitation is the inability to conduct a meta-analysis due to the considerable heterogeneity observed in both the study design and outcome measures across the included studies. This heterogeneity can make it difficult to synthesize and compare the findings of individual studies, which can compromise the accuracy and reliability of the overall conclusions.

To address this limitation, it is crucial to establish a standardized system for measuring outcomes in studies evaluating the efficacy and safety of nasal packing materials. Such a system would allow for objective comparisons of different materials and interventions, facilitating the identification of the most effective and safe options for patients. Standardization is also essential when comparing interventions between interpatient and intrapatient groups, to ensure that evaluations are not influenced by confounding factors and that the results are comparable across studies.

Furthermore, the limited number of studies available for each type of biomaterial included in this review is another critical limitation. This scarcity of data makes it challenging to draw definitive conclusions about the efficacy and safety of these materials, and highlights the need for more rigorous research in this area. Future studies with larger sample sizes and standardized protocols would provide more robust evidence to inform clinical practice and patient care. 

While the present study contributes valuable insights into the use of nasal packing materials, the identified limitations emphasize the need for further research to standardize outcome measures and study designs, increase the number of studies available for each biomaterial, and ultimately improve the quality and reliability of the evidence base. By addressing these limitations, we can advance our understanding of the use of nasal packing materials and optimize patient outcomes.

### 4.5. Future Direction

Looking ahead, there is a need for continued innovation in nasal packing. Among the currently available options, the Propel stent stands out as the most innovative due to its unique shape and mechanics, where it expands upon placement. However, the Propel stent does not have the ability to absorb blood and exudate. It would be beneficial to adapt stent technology to develop new mechanics that allow for separation between the wounded surfaces, while also incorporating an absorbable nasal pack with antimicrobial and anti-inflammatory properties to absorb exudates and blood. Additionally, the combination of two materials, such as chitosan and dextran, can be utilized to counteract each other’s disadvantages. 

Other than that, the antibacterial properties of materials can be significantly influenced by their nanoscale structure and intrinsic properties. When materials are designed at the nanoscale level, they can manifest properties such as increased surface area, enhanced reactivity, and enhanced mechanical properties [88]. These characteristics may make them more effective at killing microorganisms or inhibiting their growth. Among these materials are zinc oxide nanoparticles and silver nanoparticles. The high surface-to-volume ratio of nanoparticles enables them to interact with bacterial cell membranes and disrupt cellular function [89]. In addition, we can assume that when nasal pack containing these nanoparticles disintegrates, their diminutive size enables them to penetrate bacterial cells with greater ease, thereby enhancing their efficacy [90]. To reduce the risk of bacterial infections, silver nanoparticles have been incorporated into a variety of medical devices, including wound dressings, catheters, and implants.

## 5. Conclusions

In conclusion, the use of nasal packing materials is a critical component of endoscopic sinus surgery (ESS) to promote hemostasis and support wound healing. However, traditional packing materials such as gauze and cotton can cause pain, discomfort, and increase the risk of infection. Therefore, researchers have focused on developing new biomaterials that can improve patient outcomes, reduce complications, and enhance the safety and efficacy of ESS.

This literature review highlights the current state of knowledge regarding the efficacy and safety of different biomaterials used for nasal packing. The literature suggest that chitosan, gelatin, hyaluronic acid, and potato starch-derived materials have the potential to improve endoscopy scores and patient-reported outcomes after ESS. However, the heterogeneity of study design and outcome measures across the included studies limited the ability to draw definitive conclusions about the most effective and safe materials.

The limitations identified in this review emphasize the need for further research to standardize outcome measures and study designs, increase the number of studies available for each biomaterial, and ultimately improve the quality and reliability of the evidence base. Continued innovation in nasal packing is needed to develop new mechanics that allow for separation between the wounded surfaces, while also incorporating an absorbable nasal pack with antimicrobial and anti-inflammatory properties to absorb exudates and blood. Additionally, incorporating nanotechnology in nasal pack materials, such as zinc oxide nanoparticles and silver nanoparticles, may improve their antibacterial properties.

Overall, optimizing the use of nasal packing materials is critical to achieving successful outcomes in patients undergoing ESS or other procedures that require nasal packing. By advancing our understanding of the use of nasal packing materials and addressing the limitations identified in this review, we can improve patient care and ultimately enhance patient outcomes. 

## Figures and Tables

**Table 1:** Included studies for functional biomaterial usage following sinus surgery.

Source	Intervention/Control	Outcome Parameter	Measurement Time Point	Functional Properties
Packing Materials without Alterations				
Lorusso et al., 2021 [13]	Nasal dressing sponge^®^ (composed of PVA expanding sponge cover with a hemostatic gauze); Merocel (hydroxylated PVA)	Patient-reported outcomes: VAS (discomfort and pain); Bleeding evaluation; Nasal endoscopy: (inflammation, crusting, adhesion, and synechiae)	1 h, 6 h, 1 day, and 2 days	Hemostasis; prevent synechiae, decrease adhesion, antiproliferative properties in fibroblast
Manji et al., 2018 [28]	Silastic; gloved-Merocel (GM) spacers	Patient-reported outcomes: VAS (pain, discharge, removal pain); Nasal endoscopy: synechiae, MLK, endoscopic score	6 days, 5 weeks, and 12 weeks	Prevent synechiae, decrease adhesion, antiproliferative properties in fibroblast
Schlewet et al., (2017) [21]	Natural plant-based polysaccharides [NexPak] (modified amylopectin + hydroxyethylcellulose); synthetic polyurethane foam dressing (Nasoore).	Nasal endoscopy: degree of crusting, amount of retained implant, infection, allergic reaction to the dressing, need for postop endoscopic debridement, postop epistaxis incidence and presence of adhesion/synechia	2 and 12 weeks	Absorbable; hemostasis; prevent synechiae, decrease adhesion, antiproliferative properties in fibroblast
Jung et al., (2017) [6]	Fibrin sealant (Tisseel); Nasopore	Patient-reported outcomes: VAS (pain, facial pressure, nasal obstruction, nasal bleeding, general satisfaction); Nasal endoscopy: (grading of adhesion, bleeding, crusting, granulation, infection, and stenosis of the frontal sinus ostium)	1, 2, 4, 8, and 12 weeks	Absorbable; hemostasis
Antisdel et al., (2016) [9]	Carboxymethylcellulose (Sinufoam); potato starch-derived mucopolysaccharide hemospheres (Arista); compressible wafer made from potato starch (Nexfoam)	Patient-reported outcomes: VAS (nasal obstruction, bleeding, pain, and nasal discharge); Nasal endoscopy: (synechiae formation, mucosaledema, evidence of infection, granulation tissue formation, crusting, and time required for debridement)	1, 3, and 6 weeks	Absorbable; hemostasis; prevent synechiae, decrease adhesion, antiproliferative properties in fibroblast
Piski et al., 2016 [35]	Nasopore polyurethane nasal dressing; unpacked	Nasal endoscopy: mucosal edema, crust formation, bleeding tendency, presence of synechiae, amount of nasal discharge; the patency of the ostiomeatal complex	1, 4, and 12 weeks	Absorbable; hemostasis; prevent synechiae, decrease adhesion, antiproliferative properties in fibroblast
Park et al., (2015) [17]	Calcium alginate (Algi-Pack); carboxymethylcellulose (Sinu-Knit)	Patient-reported outcomes: VAS (scores for postoperative pain discomfort from nasal discharge and pain during packing removal and dressing change); Nasal endoscopy: adhesions, edema, and infection	1, 4, and 8 weeks	Absorbable; Hemostasis; Prevent synechiae, decrease adhesion, antiproliferative properties in fibroblast
Hsu et al., (2015) [18]	Chitosan-based bioresorbable nasal dressing (Posi-Sep X); Nasopore	Nasal endoscopy: postoperative outcome metrics included the degree of crusting, amount of retained implant, postoperative endoscopicdebridement, wound healing, epistaxis, and postoperative infection at two weeks	2 weeks	Absorbable; hemostasis; antibacterial, antiviral, antibiofilm; prevent synechiae, decrease adhesion, antiproliferative properties in fibroblast
Cho et al., 2015 [24]	Cutanplast; Spongostan	Patient-reported outcomes: VAS (nasal obstruction, postnasal drip, rhinorrhea, headache, and pain); hemostasis during nasal pack; Remaining amount of packing materials; Cost of the pack used; LKES; POSE	0, 1, 2, 7, 14, 30, 60, 90, and 180 days	Absorbable; hemostasis; antibacterial, antiviral, antibiofilm; anti-inflammatory; prevent synechiae, decrease adhesion, antiproliferative properties in fibroblast
Chan et al., 2015 [10]	Silastic stent; no stent	Patient-reported outcomes: VAS (discomfort/pain, bleeding, nasal blockage, and nasal secretion); Nasal endoscopy: edema, discharge, adhesions, and crusting.	2, 8, and 24 weeks	Prevent synechiae, decrease adhesion, antiproliferative properties in fibroblast
Matheny et al. (2014) [12]	5 mL of HA hydrogel (PureRegen Gel Sinus); carboxymethylcellulose (CMC) Sinu-Foam	Patient-reported outcomes: SNOT-20; Nasal endoscopy: VAS (ethmoid sinus inflammation, middle turbinate position, adhesion formation, polypoid tissue formation)	1, 2, 6, and 12 weeks	Drug-eluting scaffold; absorbable; anti-inflammatory; prevent synechiae, decrease adhesion, antiproliferative properties in fibroblast
Yu et al., 2014 [22]	Fibrin sealant; Merocel (hydroxylated PVA)	Patient-reported outcomes: (pain felt during packing and on removal and sensations of facial pressure/discomfort, nasal bleeding, and nasal obstruction, general satisfaction, and willingness); Nasal endoscopy: adhesion, bleeding, crusting, granulation, infection, and stenosis of the frontal sinus ostium	1, 2, 4, 8, and 12 weeks	Absorbable; prevent synechiae, decrease adhesion, antiproliferative properties in fibroblast
Raghunandhan et al., 2014 [36]	Nasopore; Merocel (hydroxylated PVA)	Patient-reported outcomes: VAS (pain, pressure, nasal block, dysphagia, sleep disturbance, postnasal drip, allergic symptoms, general satisfaction and willingness to use the material); Nasal endoscopy: nasal bleeding, mucosal injury and healing status, middle meatal synechiae, infection, granulation, adhesion, and any migration of pack over the period of follow-up	1 day, 1 week, and 4 weeks	Drug-eluting scaffold; absorbable; hemostasis; prevent synechiae, decrease adhesion, antiproliferative properties in fibroblast
Verim et al., 2014 [37]	Nasopore or Merocel; no packing	LKS; Nasal endoscopy: additional percentage of re-epithelialization; synechiae and the presence of polyps by granulation tissue, crusts; appearance of secretions, mucosal edema; presence of granulation tissue; bleeding; pain at removal; appearance of facial edema before packing removal; nasal blockage related to packing	2 weeks, 1 month, 3 months, and 6 months	Drug-eluting scaffold; absorbable; hemostasis; prevent synechiae, decrease adhesion, antiproliferative properties in fibroblast
Kastl et al., 2014 [38]	Nasopore; no packing	Duration of the inpatient stay in a double-blinded setting: side-specific postoperative bleeding, nasal breathing and feeling of pressure as well as the general parameters sleep disturbance, headaches and general well-being; which side patients considered subjectively the better was also recorded	1, 2, and 3 days	Absorbable; hemostasis; prevent synechiae, decrease adhesion, antiproliferative properties in fibroblast
Drug-Eluted Packing Materials				
Vediappan et al. (2022) [19]	Chitogel; Chitogel + Def; Chitogel + Def-GaPP; no packing	Ostium size of frontal, maxillary, and sphenoid sinuses; Patient-reported outcome: (SNOT-22, VAS (facial pain/discomfort, bleeding, nasal obstruction, and nasal secretion); LKES: (crusting, mucosa, edema, infection, granulations, and adhesion)	2, 6, and 12 weeks	Drug-eluting scaffold; absorbable; hemostasis; antibacterial, antiviral, antibiofilm; anti-inflammatory; prevent synechiae, decrease adhesion, antiproliferative properties in fibroblast
Long et al. (2022) [27]	Berberin sinonasal 103 gel (a complex gel of hyaluronic acid and berberine hydrochloride); Merocel (hydroxylated PVA) Berberine hydrochloride—treatment compound/drug	Patient-reported outcome: (VAS: symptoms of nasal obstruction, headache, facial pain/pressure, lack of smell, anterior/posterior nasal drip and overall health status); LKES (presence of polyps, discharge, for edema, scarring, and crusting); Day of hospital stays	Daily (1st–7th day)	Drug-eluting scaffold; absorbable; antibacterial, antiviral, antibiofilm; anti-inflammatory; prevent synechiae, decrease adhesion, antiproliferative properties in fibroblast
Samarei et al., 2022 [25]	TAA (2 mL of a 40 mg/mL)-impregnated bioresorbable Gelfoam sheet; Gelfoam sheet soaked with normal saline	POSE: ethmoid cavity crusting/mucosal edema/polypoid change/polyposis/secretions, middle turbinate synechia/lateralization, middle meatal antrostomy, maxillary sinus contents, sphenoid sinus, and frontal recess/sinus	1, 3, 6, 12, and 18 months	Drug-eluting scaffold; absorbable; hemostasis; anti-inflammatory; prevent synechiae, decrease adhesion, antiproliferative properties in fibroblast
Schneider et al., 2022 [39]	Frontal sinus mometasone-eluting stents (poly(L-lactide-co-glycolide) (PLG)); no packing	LM and MLM scoring; Operative findings; Patient-reported outcomes: (CRS-PRO and SNOT-22); Endoscopic severity: (polyp recurrence, MLK endoscopic severity); Mediator concentrations	6 to 12 months	Drug-eluting scaffold; absorbable; anti-inflammatory; prevent synechiae, decrease adhesion, antiproliferative properties in fibroblast
Zhang et al. (2020) [40]	PureRegen^®^ sinus gel (scHA); PureRegen^®^ sinus gel premixed with budesonide (scHA/Bu)	LKES; presences of mucus, edema, crust, scar, and polyposis	2, 4, and 12 weeks	Drug-eluting scaffold; absorbable; anti-inflammatory; prevent synechiae, decrease adhesion, antiproliferative properties in fibroblast
Rawl et al., 2019 [1]	Merocel packs wrapped in nonlatex glove material; Propel steroid-eluting stents poly(L-lactide-co-glycolide) (PLG)	Patient-reported outcomes: (SNOT-22); LK score; Middle turbinate lateralization scores	8 to 10, 20 days, 1 month, and 3 months	Drug-eluting scaffold; absorbable; anti-inflammatory; prevent synechiae, decrease adhesion, antiproliferative properties in fibroblast
Gyawali et al., 2019 [41]	Triamcinolone-soaked PVA pack; normal saline-soaked PVA pack	POSE: ethmoid cavity crusting/mucosal edema/polypoid change/polyposis/secretions, middle turbinate synechia/lateralization, middle meatal antrostomy, maxillary sinus contents, sphenoid sinus, and frontal recess/sinus; LK score: (crusting, edema, scarring/synechiae)	Week 3	Drug-eluting scaffold; prevent synechiae, decrease adhesion, antiproliferative properties in fibroblast
Hwang et al. (2018) [20]	Calcium alginate (Algi-Pack) soaked with 2 mL of triamcinolone; calcium alginate (Algi-Pack soaked with 2 mL of normal saline)	POSE: (1) middle turbinate, (2) middle meatal antrostomy, (3) maxillary sinus contents, (4) ethmoid cavity crusting, (5) ethmoid cavity mucosal edema, (6) ethmoid cavity polypoid change, (7) ethmoid cavity polyposis, (8) ethmoid cavity secretions, (9) frontal recess/sinus, and (10) sphenoid sinus	1, 4, and 8 weeks	Drug-eluting scaffold; absorbable; prevent synechiae, decrease adhesion, antiproliferative properties in fibroblast
Sow et al., 2018 [26]	Hydrocortisone-impregnated Gelfoam^®^; normal saline-impregnated Gelfoam^®^	LKES; POSE	1 week, 3 weeks, and 3 month intervals	Drug-eluting scaffold; absorbable
Ha et al. (2017) [11]	Chitosan–dextran gel (CD gel); CD gel with 1 mg/2 mL budesonide; no treatment; topical steroid cream	Ostial measurements; Nasal endoscopy: (adhesion, presence and severity, mucosal edema, granulation tissue formation, evidence of pus, and crusting) (VAS)	2 weeks, 3 months, and 12 months	Drug-eluting scaffold; absorbable; hemostasis; prevent synechiae, decrease adhesion, antiproliferative properties in fibroblast
Adriaensen et al., 2017 [5]	SinuBand with fluticasone propionate (SinuBand FP); SinuBand (without fluticasone propionate FP); 4 cm Merocel	Systemic safety (cortisol in urine); Ocular safety (intraocular pressure (IOP) and changes in lens opacity); Device-related adverse event; Nasal endoscopy (ethmoid inflammation, polyp score, synechiae/adhesion formation, Lund–Kennedy score); Patient-reported outcomes: Pain and nasal congestion score	5, 15, 30, and 60 days	Drug-eluting scaffold; absorbable; hemostasis; prevent synechiae, decrease adhesion, antiproliferative properties in fibroblast
Sabarinath et al., 2017 [2]	Polyvinyl acetate (Merocel) nasal pack soaked with triamcinolone; Merocel soaked with saline	Postoperative endoscopic grading system: (crusting, edema, polypoidal change, discharge)	1 and 2 weeks, and 1 and 3 months	Drug-eluting scaffold; anti-inflammatory
Burduk et al., 2016 [7]	Naasopore polyurethane nasal dressing impregnated with ointment (2 g of oxytetracycline and hydrocortisone); gauze strip impregnated with ointment (2 g of oxytetracycline and hydrocortisone)	Nasal endoscopy: blood crusting, edematous swelling, and epithelialization; Patient-reported outcomes: VAS	2, 10, and 30 days	Absorbable; drug-eluting scaffold; prevent synechiae, decrease adhesion, antiproliferative properties in fibroblast
Xu et al., 2016 [3]	TA-impregnated (TA group) Nasopore; saline-impregnated (control group) Nasopore	LK score; POSE; Patient-reported outcomes: SNOT-20 and Korean version of the Sniffin’ Stick (KVSS) II test; Olfactory function	1, 2, and 3 months	Absorbable; prevent synechiae, decrease adhesion, antiproliferative properties in fibroblast
Smith et al., 2016 [42]	Steroid-releasing implant; no implant	Nasal endoscopy: adhesion/scarring, polypoid edema, patency of the FSO; VAS: diameter of the FSO and the degree of inflammation; Safety assessment	Day 30	Drug-eluting scaffold; absorbable; anti-inflammatory; prevent synechiae, decrease adhesion, antiproliferative properties in fibroblast
Dautremont et al., 2014 [8]	Nasopore dissolvable spacer soaked with 2 mL of triamcinolone (40 mg/mL); no packing (postoperative placebo pill daily)	LKS; Patient-reported outcomes: SNOT-22	1 week, 3 weeks, and 2 months	Drug-eluting scaffold; absorbable; prevent synechiae, decrease adhesion, antiproliferative properties in fibroblast

LKS/LKES: Lund–Kennedy Endoscopic Score; MLK: Modified Lund–Kennedy Endoscopic Score; POSE: Perioperative Sinus Endoscopy; SNOT: Sino-Nasal Outcome Test; VAS: Visual Analogue Scale; CRS-PRO: Patient-Reported Outcome Measure for Chronic Rhinosinusitis; Chitogel: chitosan; Def: deferiprone; GaPP: gallium protoporphyrin; CD gel: chitosan–dextran gel; MPH: mucopolysaccharide hemospheres; CMC: carboxymethylcellulose; HA: hyaluronic acid; FP: fluticasone propionate; TA: triamcinolone acetonide; PVA: polyvinyl acetate; GM: gloved-Merocel; PLG: poly(L-lactide-co-glycolide).

**Table 2:** Natural biomaterials as nasal packs. Upward arrow ▲ = positive score, downward arrow ▼ = negative score, sideways arrow ◀▶ = no change/mixed effects/conflicting findings. Study quality denoted by reference column color: green = low risk bias; yellow = some concerns.

Intervention		Time Point	Study Size (Complete (Recruit))	Nasal Endoscopy Score			Patient-Reported Outcome				Other Parameter					
				LKS/LKES/MLK	POSE	Author-reported endoscopy score	SNOT-20	SNOT-22	VAS	CRS-PRO	Ostium Patency/Size	Remnant of Material	Inflammation	Bleeding	Infection	Ref.
CHITOSAN-DERIVED MATERIAL	1. Chitogel; 2. Chitogel + Def; 3. Chitogel + Def-GaPP; 4. no packing	2, 6, and 12 weeks	79 (82)	◀▶	-	-	-	▲	▲	-	▲	-	-	-	-	Vediappan et al. [19]
	1. Chitosan–dextran gel (CD gel); 2. CD gel with 1 mg/2 mL budesonide; 3. no treatment; 4. topical steroid cream	2 weeks, 3 months, and 12 months	36 (40)	-	-	▲	-	-	-	-	▲	-	-	-	-	Ha et al. [11]
	1. Chitosan-based bioresorbable nasal dressing (Posi-Sep X); 2. Nasopore	2 weeks	35 (35)	-	-	▲	-	-	-	-	-	-	-	-	-	Hsu et al. [18]
CMC-DERIVED MATERIAL	1. CMC; 2. mucopolysaccharide hemospheres (MPH); 3. compressible wafer made from potato starch	1, 3, and 6 weeks	48 (52)	-	-	▼	-	-	◀▶	-	-	-	-	◀▶	◀▶	Antisdel et al. [9]
	1. Calcium alginate; 2. CMC	1, 4, and 8 weeks	26 (27)	-	-	▼^c^ ◀▶^d^	-	-	◀▶	-	-	-	-	-	◀▶	Park et al. [17]
	1. HA hydrogel; 2. CMC	1, 2, 6, and 12 weeks	29 (30)	-	-	▼^a^ ◀▶^b^	-	-	-	-	-	-	-	-	-	Matheny et al. [12]
^a^ Overall endoscopy score compared to the other intervention; ^b^ difference in edema, crusting, or mucopurulence; ^c^ severity outcome of edema and adhesion; ^d^ edema and adhesion incidence.																
CALCIUM ALGINATE-DERIVED MATERIAL	1. Calcium alginate soaked with triamcinolone; 2. calcium alginate soaked with normal saline	1, 4, and 8 weeks	22 (22)	-	◀▶	-	-	-	-	-	-	-	-	-	-	Hwang et al. [20]
	1. Calcium alginate; 2. carboxymethyl cellulose	1, 4, and 8 weeks	26 (27)	-	-	▲^a^ ◀▶^b^	-	-	◀▶	-	-	-	-	-	◀▶	Park et al. [17]
^a^ Severity outcome of edema and adhesion; ^b^ edema and adhesion incidence.																
FIBRIN/FIBRINOGEN-DERIVED MATERIAL	1. Fibrin sealant; 2. Nasopore	1, 2, 4, 8, and 12 weeks	35 (43)	-	-	◀▶^a^ ▲^b^	-	-	◀▶^c^ ▲^d^	-	◀▶	▲	-	◀▶	◀▶	Jung et al. [6]
	1. SinuBand with fluticasone propionate (FP); 2. SinuBand (without FP); 3. Merocel pack	5, 15, 30, and 60 days	27 (30)	◀▶	-	-	-	-	▲	-	-	-	-	-	-	Adriaensen et al. [5]
	1. Fibrin sealant; 2. Merocel	1, 2, 4, 8, and 12 weeks	41 (41)	-	-	◀▶	-	-	◀▶^e^ ▲^f^	-	◀▶	▲	-	▲	◀▶	Yu et al. [22]
^a^ Mean score for adhesion, bleeding, infection, or frontal sinus ostium stenosis between sides; ^b^ mean score for crusting and granulation formation; ^c^ subjective symptoms, including pain on dressing, facial pressure, or nasal bleeding; ^d^ nasal obstruction; ^e^ pain felt during packing, facial pressure, and nasal bleeding; ^f^ pain felt during packing removal and nasal obstruction.																
GELATIN-DERIVED MATERIAL	1. TA-impregnated Gelfoam sheet; 2. Gelfoam soaked with normal saline	1, 3, 6, 12, and 18 months	104 (121)	-	-	▲	-	-	-	-	▲	-	-	-	-	Samarei et al. [25]
	1. Hydrocortisone-impregnated Gelfoam; 2. normal saline-impregnated Gelfoam	1 and 3 weeks, and 3 months	8 (8)	◀▶	◀▶	-	-	-	-	-	-	-	-	-	-	Sow et al. [26]
	1. Cutanplast; 2. Spongostan	0, 1, 2, 7, 14, 30, 60, 90, and 180 days	100 (110)	▲	▲	▲	-	-	▲	-	-	-	-	◀▶	-	Cho et al. [10]
HYALURONIC ACID-DERIVED MATERIAL	1. Berberin sinonasal 103 gel; 2. Merocel (hydroxylated PVA)	Daily (1st–7th day)	66 (66)	▲	-	-	-	-	▲	-	-	-	-	-	-	Long et al. [27]
	1. PureRegen^®^ sinus gel; 2. PureRegen^®^ sinus gel premixed with budesonide	2, 4, and 12 weeks	30 (30)	◀▶^a^ ▲^b^	-	-	-	-	-	-	-	-	-	-	-	Zhang et al. [40]
	1. HA hydrogel; 2. carboxymethylcellulose (CMC)	1, 2, 6, and 12 weeks	29 (30)	▲^c^ ◀▶^d^	-	-	-	-	-	-	-	-	-	-	-	Matheny et al. [12]
^a^ Week 12 and incidence of edema, crust, scar and poluposis; ^b^ overall endoscopic score at 3 and 6 weeks, mucus value; ^c^ overall endoscopic score, prevention of synechiae formation; ^d^ edema, crusting, mucopurulence.																
STARCH-DERIVED MATERIAL	1. Natural plant-based polysaccharides (modified amylopectin + hydroxyethylcellulose); 2. Nasopore	2 and 12 weeks	45 (45)	-	-	▲	-	-	-	-	-	-	-	-	▼	Schlewet et al. [21]
	1. Carboxymethylcellulose; 2. potato starch-derived mucopolysaccharide hemospheres (MPH); 3. compressible wafer made from potato starch	1, 3, and 6 weeks	48 (52)	-	-	▲^a^ ◀▶^b^	-	-	◀▶	-	-	-	-	◀▶	◀▶	Antisdel et al. [9]
^a^ Synechiae severity, debridement requirement, crusting, and granulation; ^b^ synechiae formation.																

LKS/LKES: Lund–Kennedy Endoscopic Score; MLK: Modified Lund–Kennedy Endoscopic Score; POSE: Perioperative Sinus Endoscopy; SNOT: Sino-Nasal Outcome Test; VAS: Visual Analogue Scale; CRS-PRO: Patient-Reported Outcome Measure for Chronic Rhinosinusitis; Def: deferiprone; GaPP: gallium protoporphyrin; CD gel: chitosan–dextran gel; MPH: mucopolysaccharide hemospheres; CMC: carboxymethylcellulose; HA: hyaluronic acid; FP: fluticasone propionate; TA: triamcinolone acetonide; PVA: polyvinyl acetate.

**Table 3:** Synthetic biomaterials as nasal packs. Upward arrow ▲ = positive score, downward arrow ▼ = negative score, sideways arrow ◀▶ = no change/mixed effects/conflicting findings. Study quality denoted by reference column color: green = low risk bias; yellow = some concerns.

Intervention		Time Point	Study Size (Complete (Recruit))	Nasal Endoscopy Score			Patient-Reported Outcome				Other Parameter					
				LKS/LKES/MLK	POSE	Author-reported endoscopy score	SNOT-20	SNOT-22	VAS	CRS-PRO	Ostium Patency/Size	Remnant of Material	Inflammation	Bleeding	Infection	Ref.
POLYURETHANE-DERIVED MATERIAL	1. Natural plant-based polysaccharides (modified amylopectin + hydroxyethylcellulose); 2. Nasopore	2 and 12 weeks	45 (45)	-	-	▼			-		-	▼	-	-	▲	Schlewet et al. [21]
	1. Fibrin sealant; 2. Nasopore	1, 2, 4, 8, and 12 weeks	35 (43)	-	-	◀▶^a^ ▼^b^	-	-	◀▶^c^ ▼^d^	-	◀▶	▼	-	◀▶	◀▶	Jung et al. [6]
	1. Nasopore; 2. unpacked	1, 4 and 12 weeks	30	-	-	▲	-	-	-	-	▲	-	-	◀▶	-	Piski et al. [35]
	1. TA-impregnated (TA group) Nasopore; 2. saline-impregnated (control group) Nasopore	1, 2, and 3 months	58 (80)	▲	▲	-	-	-	◀▶	-	-	-	-	-	-	Xu et al. [3]
	1. Nasopore impregnated with ointment (2 g of oxytetracycline and hydrocortisone); 2. gauze strip impregnated with ointment (2 g of oxytetracycline and hydrocortisone)	2, 10, and 30 days	49 (50)	-	-	▲^e^ ◀▶^f^	-	-	▲^g^ ◀▶^h^	-	-	▼	-	◀▶	▲	Burduk et al. [7]
	1. Chitosan-based bioresorbable nasal dressing (Posi-Sep X); 2. Nasopore	2 weeks	35 (35)	-	-	▼	-	-	-	-	-	▼	-	▼	▼	Hsu et al. [18]
	1. Nasopore; 2. Merocel (hydroxylated PVA)	1 day, 1 week, and 4 weeks	30	-	-	▲	-	-	◀▶	-	-	-	-	▲	◀▶	Raghunandhan et al. [36]
	1. Nasopore; 2. Merocel; 3. no packing	2 weeks, 1 month, 3 months, and 6 months	56 (58)	-	-	◀▶	-	-	-	-	-	-	-	▲	-	Verim et al. [37]
	1. Nasopore; 2. no packing	1, 2, and 3 days	47 (52)	-	-	-	-	-	▲	-	-	-	-	◀▶	-	Kastl et al. [38]
	1. TA-impregnated (TA group) Nasopore; 2. no packing (postoperative placebo pill daily)	1 week, 3 weeks, and 2 months	36	-	-	◀▶	-	-	◀▶	-	-	-	-	-	-	Dautremont et al. [8]
^a^ Mean score for adhesion, bleeding, infection, or frontal sinus ostium stenosis between sides during the follow-up period; ^b^ improvement in crusting and granulation formations; ^c^ pain on dressing, facial pressure, or nasal bleeding; ^d^ nasal obstruction; ^e^ mucosal healing and re-epithelization; ^f^ synechiae formation; ^g^ pressure; ^h^ headache.																
POLYVINYL ACETATE-DERIVED MATERIAL	1. Berberin sinonasal 103 gel; 2. Merocel (hydroxylated PVA)	Daily (1st–7th day)	66 (66)	▼	-	-	-	-	▼	-	-	-	-	-	- -	Long et al. [27]
	1. Nasal dressing sponge^®^; 2. Merocel (hydroxylated PVA)	1 h, 6 h, 1 day, and 2 days	80 (80)	-	-	-	-	-	◀▶^a^ ▼^b^	-	-	-	-	◀▶	-	Lorusso et al. [13]
	1. Merocel packs wrapped in nonlatex glove material; 2. Propel steroid-eluting stents	8 to 10 days, 20 days, 1 month, and 3 months	40 (40)	▲^c^ ◀▶^d^	-	-	-	-	◀▶	-	-	-	-	-	-	Rawl et al. [1]
	1. TA-soaked polyvinyl alcohol pack normal; 2. saline-soaked polyvinyl alcohol pack	3 weeks	58 (65)	▲	▲	-	-	-		-	-	-	-	-	-	Gyawali et al. [41]
	1. Silastic; 2. gloved-Merocel (GM) spacers	6 days, 5 weeks, and 12 weeks	48 (93)	-	-	◀▶	-	-	▲	-	-	-	-	-	-	Manji et al. [28]
	1. SinuBand with fluticasone propionate (FP); 2. SinuBand (without FP); 3. Merocel pack	5, 15, 30, and 60 days	27 (30)	-	◀▶	-	-	-	▼	-	-	-	-	-	-	Adriaensen et al. [5]
	1. TA-soaked polyvinyl alcohol pack normal; 2. saline-soaked polyvinyl alcohol pack	1 and 2 weeks, and 1 and 3 months	75	-	-	▼	-	-		-	-	-	-	-	-	Sabarinath et al. [2]
	1. Fibrin sealant; 2. Merocel	1, 2, 4, 8, and 12 weeks	41 (41)	-	-	◀▶	-	-	◀▶^e^ ▼^f^	-	-	▼	-	▼	-	Yu et al. [22]
	1. Nasopore; 2. Merocel; 3. no packing	2 weeks, 1 month, 3 months, and 6 months	56 (58)	-	-	◀▶	-	-	-	-	-	-	-	-	-	Verim et al. [37]
	1. Nasopore; 2. Merocel (hydroxylated PVA)	1 day, 1 week, and 4 weeks	30 (30)	-	-	▼	-	-	◀▶	-	-	-	-	▲	-	Ragunandhan et al. [36]
^a^ Postoperative pain; ^b^ packing removal; ^c^ middle lateralization; ^d^ LK score; ^e^ pain felt during packing, facial pressure, and nasal bleeding; ^f^ pain felt during packing removal and nasal obstruction.																
SILICONE-DERIVED MATERIAL	1. Silastic stent; 2. gloved-Merocel (GM) spacers	6 days, 5 weeks, and 12 weeks	48 (96)	-	-	◀▶	-	-	▼^a^ ◀▶^b^	-	-	-	-	-	-	Manji et al. [28]
	1. Silastic stent; 2. no stent	2, 8, and 24 weeks	36 (41)	▲	-	-	-	-	◀▶	-	-	-	-	◀▶	-	Chan et al. [10]
^a^ pain during removal; ^b^ pain prior removal and discharge.																
POLY(L-LACTIDE-CO-GLYCOLIDE) (PLG)-DERIVED MATERIAL	1. Frontal sinus mometasone-eluting Propel stents; 2. no packing	6 to 12 months	40 (52)	-	-	◀▶	-	◀▶	-	◀▶	▲	-	▲	-	-	Schneider et al. [39]
	1. Merocel packs wrapped in nonlatex glove material; 2. Propel steroid-eluting stents	8 to 10 days, 20 days, 1 month, and 3 months	40 (40)	▼	-	-	-	-	◀▶	-	-	-	-	-	-	Rawl et al. [1]
	1. Steroid-releasing implant; 2. no implant	30 days	67 (80)	-	-	▲	-	-	-	-	▲	-	▲	-	-	Smith et al. [42]

LKS/LKES: Lund–Kennedy Endoscopic Score; MLK: Modified Lund–Kennedy Endoscopic Score; POSE: Perioperative Sinus Endoscopy; SNOT: Sino-Nasal Outcome Test; VAS: Visual Analogue Scale; CRS-PRO: Patient-Reported Outcome Measure for Chronic Rhinosinusitis; FP: fluticasone propionate; TA: triamcinolone acetonide; PVA: polyvinyl acetate; GM: gloved-Merocel; PLG: poly(L-lactide-co-glycolide).

## Data Availability

The data presented in this study are available on request from the corresponding author.

## Supplementary Materials

The following supporting information can be downloaded at: https://www.mdpi.com/article/10.3390/pharmaceutics15051534/s1, PRISMA 2020 checklists; Synthesis Without Meta-Analysis (SWiM) reporting items checklist.
